# Optimizing treatment for Waldenström macroglobulinemia-associated acquired von Willebrand syndrome: a case report and literature review

**DOI:** 10.3389/fonc.2026.1766342

**Published:** 2026-03-30

**Authors:** Kai Shen, Chenlu Yang, Hongbing Ma

**Affiliations:** Department of Hematology, Institute of Hematology, West China Hospital of Sichuan University, Chengdu, China

**Keywords:** acquired von Willebrand syndrome, bortezomib, Bruton tyrosine kinase inhibitor, rituximab, Waldenström macroglobulinemia

## Abstract

Acquired von Willebrand syndrome (AVWS) is a rare bleeding disorder that is often caused by lymphoproliferative neoplasms such as Waldenström macroglobulinemia (WM). Treatment of AVWS comprises both the control of bleeding and the management of underlying diseases. As the frontline treatment of WM has rapidly evolved, the optimal regimen for AVWS secondary to WM (AVWS-WM) has yet to be elucidated. Hereby, we reported a 78-year-old male with AVWS-WM who was refractory to a rituximab-based regimen, experienced worsening bleeding when zanubrutinib was added and finally achieved a complete response to a bortezomib-based regimen. In the literature review, AVWS-WM was still rare, with only 39 cases reported. Both rituximab-based and bortezomib-based drugs are associated with a high response rate (18 out of 22 and 4 out of 5, respectively), while the Bruton tyrosine kinase inhibitor (BTKi) could result in worsening bleeding (3 out of 5) due to its inhibition of platelet function. In conclusion, both rituximab and bortezomib -based regimens are effective for AVWS-WM and regimens could be switched from one to another for refractory cases. Bruton tyrosine kinase inhibitor is not desirable front-line therapy for AVWS-WM as it could result in bleeding worsening due to its off-target inhibition of platelet function.

## 1 Introduction

Acquired von Willebrand syndrome (AVWS) is a rare bleeding disorder characterized by laboratory findings and clinical presentations similar to those of inherited von Willebrand disease (vWD) (1). The pathophysiology of AVWS is complex and involves increased degradation or clearance of circulating vWF by cell adsorption, increased proteolysis, and the presence of neutralizing antibodies (2). Lymphoproliferative neoplasms such as immunoglobulin M (IgM) monoclonal gammopathy of undetermined significance (MGUS) and Waldenström macroglobulinemia (WM) are common causes of AVWS (3). Thus, the treatment of AVWS comprises both the control of bleeding and the management of underlying diseases (4). As the frontline treatment of WM has rapidly evolved, options that include anti-CD20 monoclonal antibody-based or proteasome inhibitor (Pi)-based combinations or Bruton tyrosine kinase inhibitors (BTKis) (5). This diversity of treatment choices has made the selection of an optimal regimen for WM-associated AVWS (AVWS-WM) more difficult. Thus, we report a patient with AVWS-WM who was refractory to a rituximab-based regimen, experienced worsening bleeding when BTKi was added and finally achieved a complete response to a bortezomib-based regimen. We also reviewed the literature and proposed an algorithm for the treatment selection of AVWS-WM.

## 2 Case report

A 78-year-old male patient was evaluated in our center for hemoptysis and gingival bleeding in December 2022. He had no signs of bruising, epistaxis, or gingival bleeding upon admission. He was diagnosed with rectal cancer which was successfully removed 6 years ago without relapse at the regular follow-up. He had no abnormal coagulation parameters documented during the previous procedure. His coagulation screening test revealed prolongation of activated partial thromboplastin time (APTT) at 57.5 second (s) which could not be fully corrected by mixing with normal plasma. His factor VIII coagulant activity (FVIII:C) was 4.4%. Thus, he was first suspected of having acquired hemophilia A and treated with prednisone at 1 mg/kg body weight. The patient’s APTT fluctuated between 40 and 70 s (ratio: 1.20 ~2.11) afterwards, with episodes of easy bruising and gum bleeding.

In August 2023, he was hospitalized again due to the exacerbation of gum bleeding. Screening for vWD was arranged. First, the ristocetin-induced platelet aggregation (RIPA) rates were 4.28%, 3.7% and 52.64% with ristocetin concentrations of 0.5, 1.0 and 1.5 mg/mL, respectively. His vWF antigen (vWF: Ag) was 22.5%. In contrast, the vWF glycoprotein Ib-mutant activity (vWF: Act) was greater than upper normal limit, suggesting the presence of antibody interference. Thus, he was diagnosed with acquired von Willebrand syndrome (AVWS). For etiological screening, serum electrophoresis and immunofixation detected M protein (2.2 g/L) of IgM κ type. Immunofixation further confirmed the presence of monoclonal IgM κ. The serum IgM concentration was 12.7 g/L (normal range, 0.7–2.2 g/L). He had no hyperviscocity related symptoms or lymphosplenohepatomegaly in physical examination. Bone marrow (BM) aspiration with flow cytometry (FCM) revealed the presence of both monoclonal B lymphocytes and monoclonal plasma cells. Therefore, he was diagnosed with WM. Plasmapheresis was refrained considering the bleeding risk and no hyperviscocity present.

Consequently, he was treated with rituximab 375 mg/m^2^ weekly for 4 weeks followed by monthly infusion of rituximab for 2 months to control WM and exterminate the antibody against vWF. However, the APTT fluctuated around 50–60 s. Thus, 160 mg of zanubrutinib was added. Nevertheless, only 2 weeks after using zanubrutinib, the patient experienced widespread bruising with gums bleeding and zanubrutinib was immediately stopped. Then, his treatment was switched back to rituximab-based immunochemotherapy (rituximab, cyclophosphamide and dexamethasone). During that period, his lowest APTT was 41.6 s. A weak monoclonal IgM band could still be identified by IFE.

**Figure 1:**
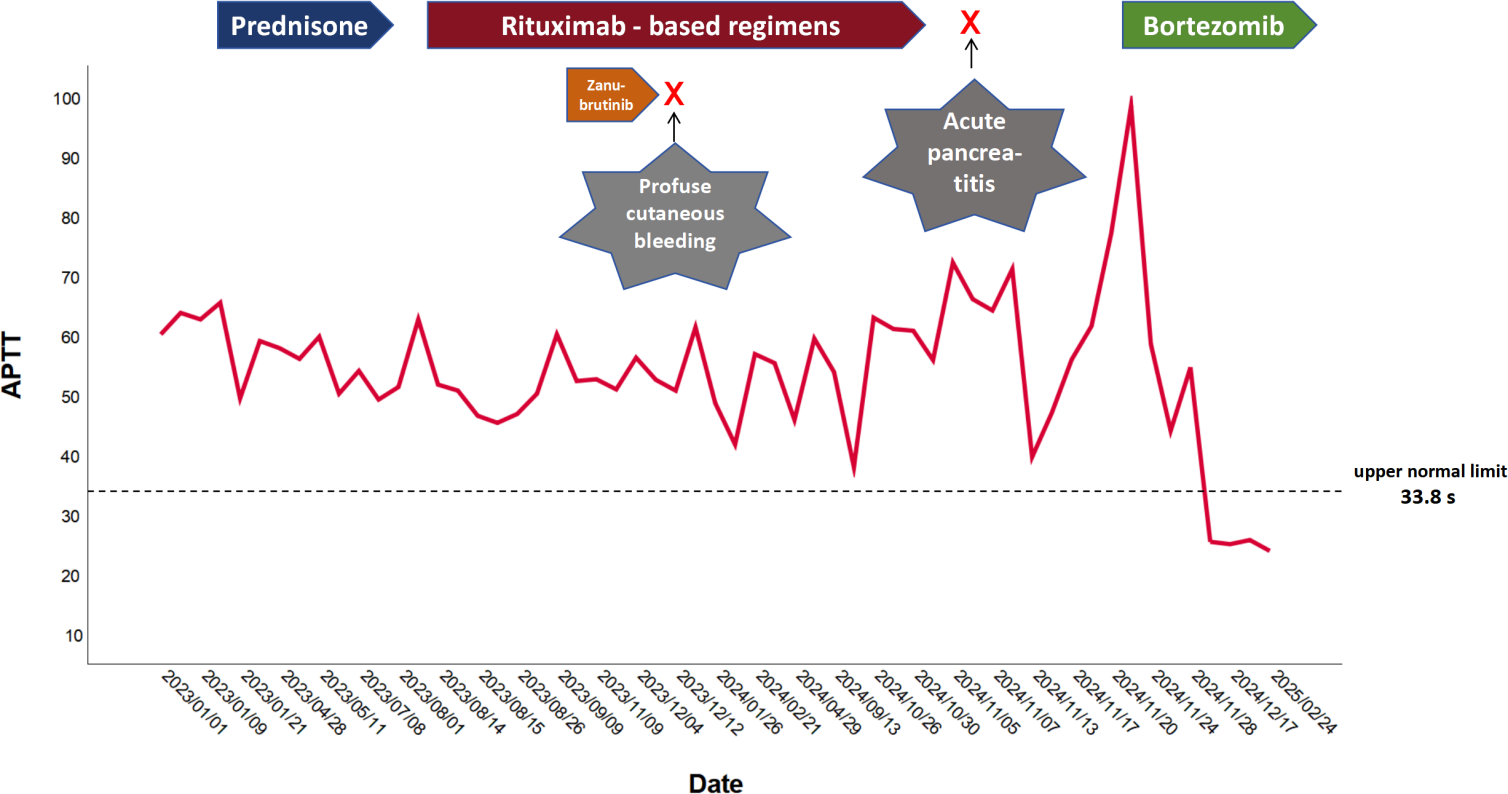
APTT trend reflected the response to different regimens in our patient.

In October 2024 he was hospitalized again for acute pancreatitis and acute cholecystitis secondary to gallstones, and rituximab therapy was discontinued. After that, his APTT peaked at 98.7 s. After his acute pancreatitis was alleviated, VD regimen (bortezomib given intravenously at 1.3 mg/m2 weekly and dexamethasone given orally at 20 mg weekly) was attempted for his AVWS. Only after the first 4-week cycle of VD regimen, the patient’s APTT restored to 25.3 s. Moreover, his vWF: Ag and vWF: Act improved to 41.4% and 71.1%, respectively. This positive response was maintained at the 3-month follow-up, and a recent IFE revealed no monoclonal IgMκ band. The response of each treatment in terms of APTT for this patient is summarized in **Figure 1**.

PubMed search for articles was performed up to May 2025 without an initial date. Articles with descriptions of tumor-targeted treatment and the clinical response to AVWS were included. Other articles concerning only hemostatic drugs or plasmapheresis for AVWS were excluded. This selection strategy ultimately identified 15 eligible articles for analysis (1, 6–19). These articles described 39 cases (**Supplementary Table 1**). A total of 46 therapeutic regimens and their responses were collected from these patients. The median age was 70 years (range, 35–90). The male-to-female ratio was 1.3:1. The most common manifestations are mucocutaneous bleeding (epistaxis, bruising) and bleeding after invasive procedures such as tooth extraction. Prolonged APTT and reduced factor VIII activity are commonly described as the first indicators of coagulation disorders. The median FVIII:C was 29% (range, 9–57%). vWF: Ag (median, 24%; range, 0.5-47%) and vWF: Rco (median, 17; range, 0.5-46%) were also low. In the multimer analysis, 2 patients had loss of high-molecular-weight (HMW) multimers, 2 patients had blurred structures, and 2 patients had normal bands. The median IgM paraprotein level was 45.2 g/L (range, 3–84 g/L). The tumor-directed treatment regimens include chemotherapy, rituximab or rituximab plus chemotherapy, and BTKi-based and proteasome inhibitor-based methods. The responses to each protocol are summarized in **Table 1**. Among the patients with bleeding manifestations, 22 received rituximab-based regimens, and 18 were responsive in terms of the resolution or improvement of bleeding symptoms. However, 3 out of the 5 patients who received BTKis (ibrutinib and zanubrutinib) reported worsening bleeding. 5 out of the 6 patients who received Pi-based regimens were responsive.

**Table 1:** Treatment regimens for AVWS-WM and their efficacy from the literature.

Regimens	Number	Response rate for AVWS	Notes
Rituximab	4	75%	1 case had no bleeding symptoms
BR or BRD	7	71.4%	1 case had IgM flare with bleeding
R-COP	5	100%	
R-Clb	1	100%	
RCD	6	100%	
FR	1	0%	
BTKi-based	6	40%	1 case had IgM flare with bleeding due to rituximab; 2 cases experienced worsening bleeding; 1 case had no bleeding symptoms
Bortezomib-based	5	80%	
CaRD	1	100%	
Lenalidomide	1	100%	
Idelalisib	1	100%	
Chemotherapy	8	87.5%	

BR, bendamustine and rituximab; BRD, bendamustine, rituximab and dexamethasone; R-COP, rituximab, cyclophosphamide, vincristine and prednisone; R-Clb, rituximab and chlorambucil; RCD, rituximab, cyclophophamide and dexamethasone; FR, fludaribine and rituximab; BTKi, bruton tyrosine kinase inhibitor; CaRD, carfilzomib, rituximab and dexamethasone.

## 3 Discussion

AVWS is associated primarily with lymphoid neoplasms, with WM as the most common cause. In WM, vWF can be degraded due to autoantibody destruction, increased shear stress due to hyperviscosity, or sequestered due to adsorption onto malignant cells, leading to AVWS-WM (1, 20). Thus, screening for AVWS is necessary in WM patients with bleeding manifestations. Nevertheless, the true prevalence of AVWS in WM patients is still unclear. In a WM cohort of 2210 patients, only 77 patients (3.4%) had vWF testing performed, and 17 patients (22% of those tested; 0.8% of the total) had confirmed AVWS-WM (19). AVWS was also reported in IgM-MGUS which was defined as a pre-WM condition with serum IgM monoclonal protein of any size and without bone marrow lymphoplasmacytic infiltration. AVMS secondary to IgM-MGUS has been categorized into monoclonal gammopathies of clinical significance (MGCS) with similar diagnostic and therapeutic consideration (21).

The main symptom of AVMS is mucocutaneous bleeding, which is consistent with the defect in primary hemostasis. The most common method for identifying an inhibitor against vWF is based upon APTT correction test, followed by the measurement of both vWF: Ag and vWF: Act (22). RIPA as a surrogate still showed subnormal aggregation at the standard testing concentration (1.5 mg/mL), which was highly suggestive of vWD. The abnormally high level of vWF:activity suggested the presence of antibody interference. Antibodies can interfere with vWF binding to glycoproteins Ib or collagen), which explains the different results for the abnormally high level of vWF: Act (23, 24).

Given that treatment of the underlying etiology is the only possible cure for AVWS, WM-directed systemic therapy is needed for AVMS-WM. As clinical cases with no efficacy are less likely to be reported, the efficacy of the current protocols may be overestimated. This explains the high response rate in traditional chemotherapy patients. According to the literature review, the use of rituximab as a single agent or rituximab-based chemotherapy is still the most commonly used regimen for AVMS-WM. Nevertheless, some patients may experience IgM flares and worsening bleeding symptoms due to the accelerated clearance of VWF in high sheer force conditions, such as hyperviscosity (25). Thus, cautious monitoring of IgM levels, bleeding manifestations and coagulation parameters are warranted when rituximab is used on the front line, especially for those with high IgM levels at diagnosis. Rituximab×4-week regimen is recommended for IgM MGUS rather than WM (26). Rituximab combined with bendamustine (BR) or cyclophosphamide and/or dexamethasone (RC or RCD) could further improve the response rate and progression-free survival of WM (27, 28). Currently, the BR regimen is one of the Mayo Clinic preferred induction protocols for newly diagnosed WM because of its ease of use and low rates of nonhematologic adverse events (5). However, for senile or frail patients such as our patient, complications such as infection after immunochemotherapy may cause treatment discontinuation and the recurrence of bleeding events. Thus, less toxic regimens are needed for unfit patients.

In clinical trials, first- and second-generation BTKi therapies have demonstrated a high overall response rate of over 90% in WM patients (29–31). Although there have been only limited cases of BTKi use in AVWS-WM, worsening of bleeding symptoms seems to be a common event because of the off-target antiplatelet effects of BTKi due to the inhibition of platelet collagen receptor GP VI downstream signaling (32). GP VI is responsible for platelet activation and aggregation in response to collagen exposure. Although comparative studies have shown that second-generation BTKi acalabrutinib and zanubrutinib are less potent inhibitors of GPVI-mediated platelet aggregation than ibrutinib (33), zanubrutinib led to the worsening of bleeding in AVWS-WM in our case and in the literature (19). This may be the result of the dual inhibition of platelet function by zanubrutinib and vWF antibodies. Thus, considering the risk of bleeding, the BTKi is not a desirable choice for AVWS-WM patients with uncontrolled diseases. Nevertheless, BTKis may play a role in maintenance treatment.

Proteosome inhibition is another important choice for WM management. Bortezomib has been shown to have high levels of activity in patients with relapsed WM, with response rates ranging from 81% to 96% (34, 35). CXCR4 mutation does not lower the response rate to bortezomib. In our study, bortezomib induced a fast response, as demonstrated by the normalization of APTT and vWF: Ag. Moreover, 5 out of the 6 AVWS-WM patients treated with bortezomib-containing regimens achieved good responses. Thus, bortezomib-based regimens combined with either rituximab (VRd) or cyclophosphamide (VCd) could also be a reasonable choice for front-line therapy of AVWS-WM. The next-generation proteasome inhibitors carfilzomib and ixazomib have been reported to be effective for treating AVWS-WM in sporadic case reports (7, 19). Thus, they could be considered in the latter line of therapy.

**Figure 2:**
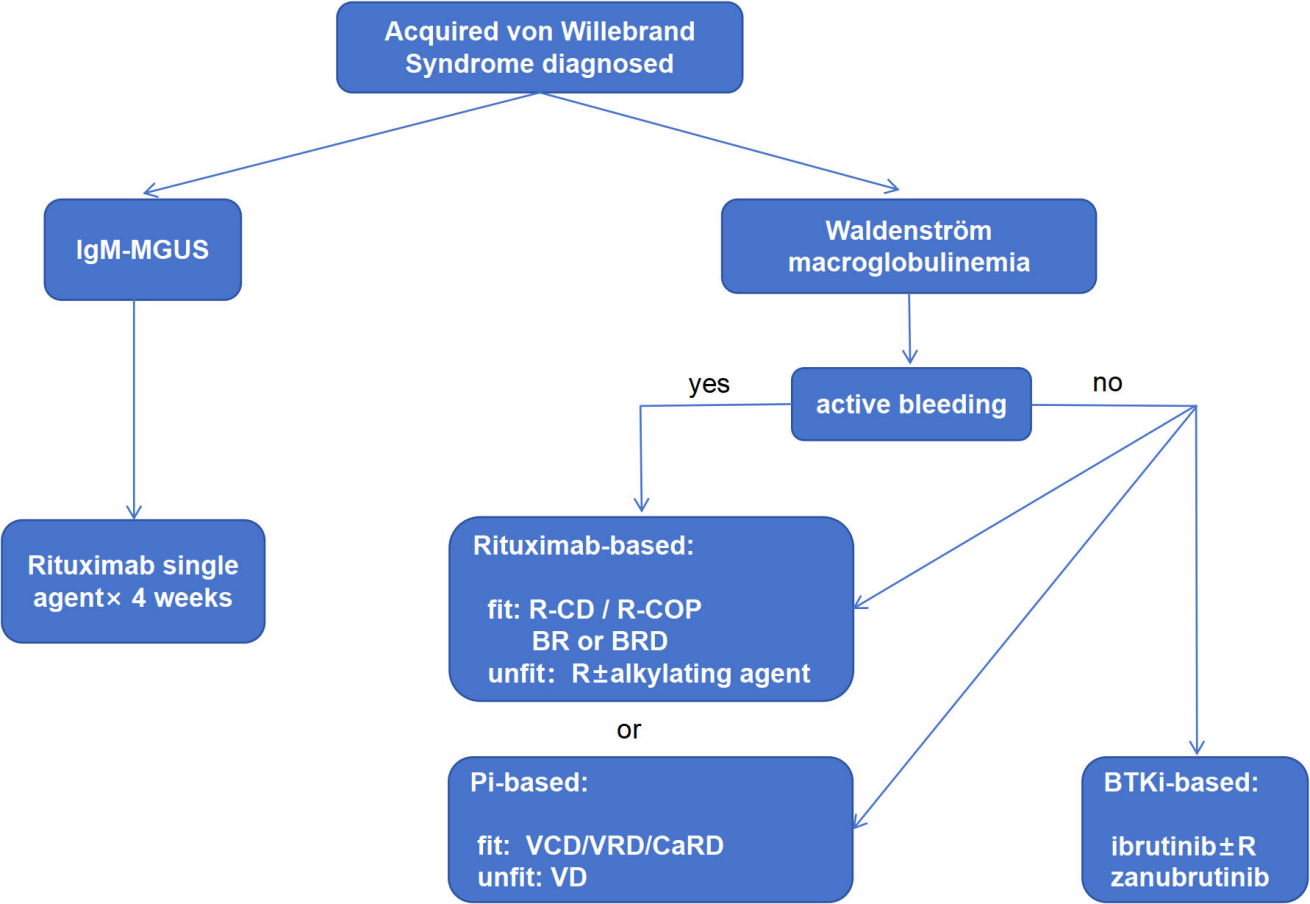
Proposed algorithm of the treatment selection for AVWS-WM. Both rituximab-based and Pi-based therapies can be used in the frontline setting and treatment regimen could be switched from one to another when the disease is refractory. Rituximab as a single agent could only be considered when the background disease is IgM-MGUS which is a pre-WM condition characterized by the presence of monoclonal IgM but no lymphoplasmacytic infiltration in the bone marrow or any evidence of lymphoma. BTKi is not recommended to patients with active bleeding. When bleeding is worsened after BTKi exposure, BTKi should be stopped and alternative therapy should be considered.

On the basis of the current evidence from a literature review, we propose an algorithm for the treatment of AVWS associated with monoclonal IgM (**Figure 2**). Generally, both rituximab-based and Pi-based therapies targeting WM can be used for AVMS-WM. BTKis can only be used for nonbleeding patients. Once a patient has worsening bleeding after BTKi exposure, BTKi should be stopped, and alternative treatment should be considered. Rituximab monotherapy is only recommended when the patients had serum monoclonal IgM but no evidence of bone marrow involvement with lymphoplasmacytic lymphoma (IgM-MGUS). Furthermore, pharmaceutical approval, drug availability and reimbursement policy are also influencing the selection and sequencing of therapies for AVMS-WM in different countries.

In summary, AVWS-WM is a rare bleeding disorder likely caused by monoclonal IgM autoantibodies against vWF. When laboratory findings suggest vWD in a patient with a negative history of bleeding diathesis, the possibility of AVWS should be explored. WM-directed systemic therapy is needed in AVMS-WM for the clearance of autoantibodies. Plasmapheresis should be reserved considering the risk of bleeding. The limited data from our case and literature review suggest that both rituximab- and bortezomib -based therapies are effective. Although rituximab-based regimens are more often chosen, bortezomib could be used for rituximab-refractory cases. BTKis could lead to worsening of bleeding in AVWS-WM due to the dual inhibition of platelet function by both BTKis and autoantibodies. Thus, the BTKi is not suitable as a frontline treatment for patients at risk of bleeding.

## Data Availability

The raw data supporting the conclusions of this article will be made available by the authors, without undue reservation.
