# Supplementary material for: Optimizing treatment for Waldenström macroglobulinemia-associated acquired von Willebrand syndrome: a case report and literature review
Source: Front Oncol. 2026 Mar 30;16:1766342. doi: 10.3389/fonc.2026.1766342 (PMC13070815; doi:10.3389/fonc.2026.1766342)
Supplement: Supplementary file 1 [file Table1.docx]

Supplement table 1. AVWS-WM cases in the literature review.

| No | Age | Sex | Bleeding manifestations | M protein(g/L) | APTT(s) | FVIII:C  (%) | vWF:Ag  (%) | vWF:Rco  (%) | Multimeric profile | Regimen | Bleeding symptoms response | WM response | Reference |
| --- | --- | --- | --- | --- | --- | --- | --- | --- | --- | --- | --- | --- | --- |
| 1 | 71 | M | Bleeding aftertooth extraction | - | prolonged | 55 | 35 | 30 | blurred structure | R-COP | resolution | - | [6] |
| 2 | 52 | F | Menorrhagia,epistaxis, fundus bleeding | - | prolonged | 26 | 26 | 32 | - | R-COP | resolution | - |  |
| 3 | 61 | F | epistaxis | 3 | - | 34 | 18 | <20 | absence of HMW-VWF | CaRD | resolution | - | [7] |
| 4 | 73 | M | wound bleeding, epitaxis, GI bleeding | - | - | - | 31 | 13 | - | BR | continued | CR | [8] |
|  |  |  |  |  |  |  |  |  |  | lenalidomide | resolution | CR |  |
| 5 | 83 | M | hematuria | 35 | 56 | 33 | 10 | 35% | - | chlorambucil | resolution | - | [9] |
| 6 | 75 | F | epistaxis | - | - | 18 | <10 | <10 | - | chlorambucil | resolution | - |  |
| 7 | 73 | - | epistaxis | - | - |  | 37 | 28 | - | chemotherapy | resolution | PR | [10] |
| 8 | 35 | - | - | - | - |  | 27 | 17 | - | chemotherapy | resolution | PR |  |
| 9 | 50 | - | rectal hemorrhage | - | - |  | 24 | 15 | - | chemotherapy | resolution | CR |  |
| 10 | 90 | - | - | 84 | prolonged | 57 | 26 | 39 | - | R-COP | - | - | [11] |
|  |  |  |  |  |  |  |  |  |  | VMP | resolution | VGPR |  |
| 11 | 70 | - | - | 72 | prolonged | 21 | 28 | 27 | - | R-COP | - | - |  |
|  |  |  |  |  |  |  |  |  |  | VMP | resolution | VGPR |  |
| 12 | 79 | - | - | 39 | prolonged | 44 | 37 | 41 | normal | idelalisib | resolution | VGPR |  |
| 13 | 65 | M | epistaxis, bruising | 44 | 44 | 20 | 16 | 11 | - | R-COP | resolution | PR | [12] |
| 14 | 70 | M | epistaxis, bruising | 50 | 58.6 | 14 | 16 | 5.2 | normal | VTD | resolution | PR | [13] |
| 15 | 70 | M | gingival bleeding | 8.8 | prolonged | 40 | 18 | 29 | - | BR | resolution | PR | [14] |
| 16 | 63 | F | epistaxis and ecchymosis | 4 | 40 | 47 | 47 | 46 | - | VRD | continued | PR | [15] |
| 17 | 75 | F | rectal bleeding after surgery, hemarthrosis | 50 | prolonged |  | 14 | 15 | - | RCD | resolution | PR | [16] |
|  |  |  |  |  |  |  |  |  |  | Ibrutinib | resolution | VGPR |  |
| 18 | 54 | M | gingival bleeding | 45.2 | 68 | 9 | 0.7 | 0.5 | absence of VWF multimers | VCD | resolution | VGPR | [17] |
| 19 | 77 | M | none | 77.4 | 47.8 | 16 | 14 | 14 | - | R-Clb | continuation | SD | [18] |
| 20 | 67 | M | epitaxis | 50.7 | 60 | 24 | 15 | 9 | normal | RCD | resolution | PR |  |
| 21 | 77 | F | bleeding after dental implants | 52 | 84 | 43 | 35 | 26 | - | RCD | resolution | PR |  |
| 22 | 63 | M | retroperitoneal haematoma, epistaxis, bruising | 42.5 | 55.3 | 29 | 23 | 14 | blurred structure | FR | continued | PR | [19] |
| 23 | - | - | cutaneous bleeding, intraabdomi-l hematoma after CT-guided biopsy | - | - | - | - | - | - | rituximab | resolution | - | [20] |
|  |  |  |  |  |  |  |  |  |  | zanubrutinib-->acalabrutinib | resolution | - |  |
| 24 | - | - | no bleeding | - | - | - | - | - | - | RCD | no bleeding symptoms | PR |  |
| 25 | - | - | recurrent GI bleeding | - | - | - | - | - | - | Rituximab | resolution | SD |  |
| 26 | - | - | wrist hematoma | - | - | - | - | - | - | BRD | IgM flare with bleeding requiring IVIG, Factor VIII/ VWF and therapy change | MR |  |
| 27 | - | - | no bleeding | - | - | - | - | - | - | rituximab | no bleeding symptoms | PR |  |
|  |  |  |  |  |  |  |  |  |  | ibrutinib+ixazomib | no bleeding symptoms | - |  |
| 28 | - | - | epistaxis, subarachnoid and reti-l hemorrhage | - | - | - | - | - | - | RCD | improvement of bleeding | MR |  |
| 29 | - | - | retimal hemorrhage, epistaxis | - | - | - | - | - | - | BR | resolution | PR |  |
| 30 | - | - | epistaxis | - | - | - | - | - | - | RCD | resolution | MR |  |
| 31 | - | - | retinal hemorrhage, epistaxis | - | - | - | - | - | - | ibrutinib+ixazomib | IgM flare with bleeding requiring therapy change | PR |  |
| 32 | - | - | bruising, epistaxis | - | - | - | - | - | - | ibrutinib-Rituximab | worsewing | PR |  |
| 33 | - | - | GI bleeding, epistaxis, cutaneous bleeding | - | - | - | - | - | - | BR | improvement | PR |  |
|  |  |  |  |  |  |  |  |  |  | zanubrutinib | worsening | - |  |
| 34 | - | - | epistaxis | - | - | - | - | - | - | cladribine | improvement | PR |  |
| 35 | - | - | epistaxis | - | - | - | - | - | - | fludarabine | resolution | PR |  |
| 36 | - | - | epistaxis, GI bleeding | - | - | - | - | - | - | chlorambucil | resolution – recurrence of bleeding symptoms post- therapy | - |  |
| 37 | - | - | epistaxis, hematuria | - | - | - | - | - | - | rituximab | resolution – recurrence of bleeding symptoms post- therapy | PR |  |
| 38 | - | - | recurrent knee hemarthrosis, retinal hemorrhage, thigh hematoma | - | - | - | - | - | - | BR | resolution | PR |  |
| 39 | - | - | post-menopausal vaginal bleeding and gum bleeding | - | - | - | - | - | - | BR | resolution | PR |  |
